# Supplementary material for: MetaRibo-Seq measures translation in microbiomes
Source: Nat Commun. 2020 Jun 29;11:3268. doi: 10.1038/s41467-020-17081-z (PMC7324362; doi:10.1038/s41467-020-17081-z)
Supplement: Supplementary file 10 — Supplementary Data 7 [file 41467_2020_17081_MOESM10_ESM.zip › File2/Confidence_High_Taxonomy/387611_out.krona.html]

Javascript must be enabled to view this page.

members
magnitude
magnitudeUnassigned
count
unassigned
taxon
rank

387611\_out

6

superkingdom
6
2

1239
6
phylum

186801
6
class

order

SRS014979\_contig\_number\_27167SRS053335\_contig\_number\_9868
2
186802
6

2039302
4
genus

species

SRS013965\_contig\_number\_40714SRS014923\_contig\_number\_18653SRS015217\_contig\_number\_31763SRS104400\_contig\_number\_5462
4
1981510
